# Exploring the quality of life issues in people with retinal diseases: a qualitative study

**DOI:** 10.1186/s41687-017-0023-4

**Published:** 2017-09-21

**Authors:** Mallika Prem Senthil, Jyoti Khadka, Jagjit Singh Gilhotra, Sumu Simon, Konrad Pesudovs

**Affiliations:** 1 NHMRC Centre for Clinical Eye Research, Flinders University of South Australia, Adelaide, South Australia 5001 Australia; 2 University of Adelaide, Adelaide, South Australia 5005 Australia

**Keywords:** Hereditary retinal diseases, Acquired retinal diseases, Quality of life, Patient-reported outcome measures, Qualitative, Interviews

## Abstract

**Background:**

The lack of an appropriate retina-specific patient-reported outcome instrument restricts the understanding of the full impact of hereditary retinal diseases and other less common but potentially blinding acquired retinal diseases such as, vascular occlusions, epiretinal membrane, macular hole, central serous retinopathy and other vitreoretinopathies on quality of life. This study aims to explore the quality of life issues in people with hereditary retinal diseases and acquired retinal diseases to develop disease-specific patient-reported outcome instruments.

**Methods:**

A qualitative research methodology to understand the lived experiences of people with retinal diseases was carried out. Data were collected through semistructured interviews. The coding, aggregation and theme development was carried out using the NVivo −10 software.

**Results:**

Seventy-nine interviews were conducted with participants with hereditary retinal diseases (*n* = 32; median age = 57 years) and acquired retinal diseases (*n* = 47; median age = 73 years). We identified nine quality of life themes (domains) relevant to people with retinal diseases. Difficulty in performing important day-to-day activities (activity limitation) was the most prominent quality of life issue in the hereditary retinal diseases group whereas concerns about health, disease outcome and personal safety (health concerns) was the most prominent quality of life issue in the acquired retinal diseases group. Participants with hereditary retinal diseases had more issues with social interaction (social well-being), problems with mobility and orientation (mobility), and effect on work and finance (economic) than participants with acquired retinal diseases. On the contrary, participants with acquired retinal diseases reported more inconveniences (conveniences) than participants with hereditary retinal diseases, which were mostly attributed to treatment. Participants with hereditary retinal diseases were coping better compared to participants with acquired retinal diseases.

**Conclusions:**

Our study found that participants with both hereditary and acquired retinal diseases are living with myriad of disease-specific quality of life issues. Many of these issues are completely different and unique to each disease group. Hence, these group of diseases would need separate patient-reported outcome instruments to capture the disease-specific quality of life impacts.

**Electronic supplementary material:**

The online version of this article (10.1186/s41687-017-0023-4) contains supplementary material, which is available to authorized users.

## Background

Quality of life is severely compromised in people with major blinding retinal diseases such as age related macular degeneration and diabetic retinopathy [1–6]. Very little is known about quality of life impacts in people with other vitreoretinal diseases (i.e. hereditary degenerations, vascular occlusions, macular hole, epiretinal membrane and other vitreoretinopathies). Research exploring the impact of other vitreoretinal diseases on quality of life has been restricted by the lack of appropriate patient-reported outcome instruments. To date, only a few retina-specific patient-reported outcome instruments are available for other vitreoretinal diseases [7–9]. Moreover, these patient-reported outcome instruments have undergone only basic validation procedures and the content coverage is limited to measuring only a few quality of life domains (mostly activity limitation) [10]. Moreover, there are no patient-reported outcome instruments developed for vascular occlusions, macular hole and epiretinal membrane. Quality of life impacts in these retinal conditions are mostly assessed using non-disease-specific patient-reported outcome instruments (ophthalmic instruments that have been originally developed for other eye disease/s) and generic instruments (instruments developed for non-ocular diseases) [11–17]. In some contexts, the generic and non-disease-specific instruments (e.g., Health Utilities Index used as an outcome measure on macular degeneration and cataract surgery) have been shown to be responsive, [18–22] but they do not contain disease-specific items and hence may be less sensitive in assessing the quality of life impacts of people with specific diseases compared to the disease-specific instruments [10]. The lack of an appropriate retina-specific patient-reported outcome measure restricts our understanding of the full impact of these vitreoretinal diseases and their treatment on quality of life. Understanding patients’ perspective is critical as new treatment modalities such as anti-vascular endothelial growth factor intravitreal injections and gene therapy are gaining momentum especially for vascular occlusive diseases and hereditary degenerations.

The commonly occurring retinal diseases in terms of the number of patients affected such as age related macular degeneration, diabetic retinopathy, and retinal detachment deserve to have separate patient-reported outcome instruments. However, it is not feasible to develop separate patient-reported outcome instruments for the less common vitreoretinal diseases such as hereditary degenerations, vascular occlusions, and other vitreoretinopathies. Nevertheless, these less common retinal diseases do need more targeted and specific patient-reported outcome instrument/s that could accurately measure quality of life impact and be sensitive to the treatment outcomes. A way forward would be to lump or split these vitreoretinal diseases into groups based on similar quality of life issues to develop group-specific patient-reported outcome instruments.

We are developing technologically advanced patient-reported outcome measures in the form of item banks implemented via computerized adaptive testing for other vitreoretinal diseases. Item banks are a large collection of calibrated items that measure an underlying latent trait (e.g., functional limitation, emotional well-being) [23]. The computerized adaptive testing selects the items from the item banks that closely match the participant’s ability level. The computerized adaptive testing iteratively administers items based on the participant’s responses to previous questions and therefore the computerized adaptive testing requires very few items to provide a precise and accurate assessment of patient-reported outcome measures [24, 25]. Item banking implemented via computerized adaptive testing can provide solutions to the issues associated with the traditional paper-and pencil based questionnaires which are static, have limited applicability to population, outdated and do not provide a holistic assessment of quality of life [23, 26–29]. Item banks have been successfully developed and implemented in other fields of health care [30–32]. Item banks have been developed for other ocular diseases such as glaucoma, age related macular degeneration and diabetic retinopathy [1, 33, 34]. This study aims to qualitatively explore the quality of life issues of people with other vitreoretinal diseases to develop group-specific item banks.

## Methods

The qualitative theoretical framework that was used to explore the quality of life issues of people with retinal diseases was phenomenology. Phenomenology is concerned with in-depth understanding of the participants’ lived experiences and the meanings that the participants perceive of those experience [35]. A non-probability, convenience sampling technique was used to recruit 79 participants with different retinal diseases. For adequate number of participants’ recruitment, we categorized the vitreoretinal diseases into hereditary retinal diseases and acquired retinal diseases. This was done because hereditary retinal diseases and acquired retinal diseases differ vastly in terms of the nature, age of onset, laterality, and progression of the disease. Hereditary retinal diseases tend to occur at an early age; they are mostly bilateral and progressive in nature. On the other hand, acquired retinal diseases have a late onset and mostly unilateral to begin with [36–39]. Hereditary retinal diseases includes retinitis pigmentosa, macular dystrophies, choroidal dystrophies, and other hereditary vitreoretinopathies. Acquired retinal diseases group includes relatively less common but potentially blinding retinal diseases such as vascular occlusions, macular hole, epiretinal membrane, and other rare vascular disorders. As the aim of this study was to develop item banks for other vitreoretinal diseases, we excluded people with major blinding retinal conditions such as age related macular degeneration, diabetic retinopathy and retinal detachment.

Participants for the hereditary retinal diseases group were recruited from welfare and charity organizations (The Royal Society for the Blind and Retina Australia) through emails and flyers. Participants for the acquired retinal diseases group were recruited from the retina clinics of two major metropolitan public health care facilities (The Royal Adelaide Hospital and The Queen Elizabeth Hospital). Clinical records were used to identify potential participants who were then approached to discuss their possible involvement in the study. Participants for the hereditary retinal diseases group were recruited from charity organizations and not hospitals because hereditary retinal diseases are a rare group of disorders, which are mostly untreatable and hence not commonly seen in hospitals. Hereditary retinal diseases are progressive in nature that ultimately cause blindness and participants with hereditary retinal diseases are more likely to join organizations to seek information and support. They are also more likely to take part in research. On the contrary, acquired retinal diseases are relatively common retinal conditions that are mostly treatable and so commonly seen in hospitals. Hence, participants for the acquired retinal diseases group were recruited from retina clinics. Participants who were interested in participating in the study were sent out an information pack with an invitation letter, participation information sheet, consent form and a demographic form. Upon receiving the consent form, the participants were contacted through telephone to organize a date and time for the interview. Informed consent was obtained from all individual participants included in the study. All the participants were recruited to a single in-depth interview. Ethical approval was obtained from the Southern Adelaide Clinical Human Research Ethics Committee and the study adheres to the Tenets of Declaration of Helsinki.

A semi-structured interview guide was developed from existing literature (pre-existing patient-reported outcome instruments and qualitative studies) and was validated by a panel of experts (MP, JK, KP, GS & SS). The authors JK and KP are internationally recognised experts in patient-reported outcome development and validation. They are also optometrists with extensive clinical experience. The authors MP, GJ, and SS are ophthalmologists with clinical experiences in retinal diseases. The authors MP, JK and KP are located at Flinders University and the authors GJ and SS are located at The University of Adelaide.

The aim of this semi-structured interview guide was to include questions that would help to uncover all aspects of quality of life (physical, mental, and social) (Additional file 1). One of the authors (MP) did the interviews either by face-to face or over the telephone. All interviews were audio recorded and transcribed. There was no predetermined number of participants to be recruited at the start of the study. The sampling process continued until the emerging theoretical categories were saturated.

### Data analysis

**Fig. 1:**
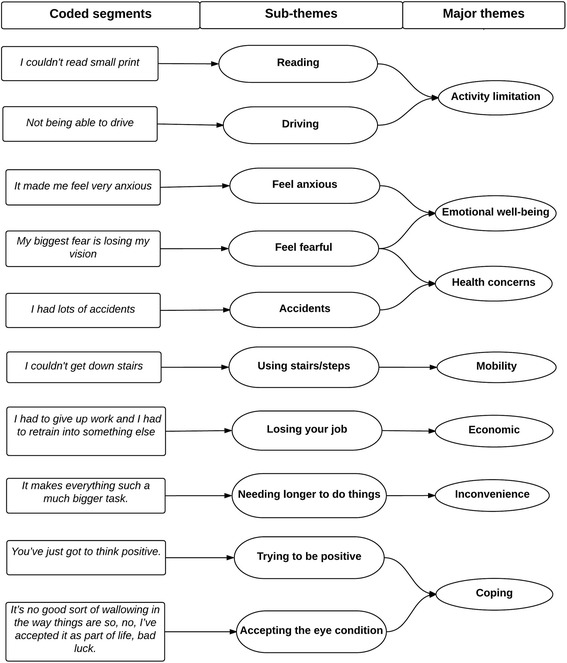
Process of data analysis and themes development

The data analysis occurred after the data collection was complete. An open coding technique was adopted to analyze the textual data. In an open coding technique, the data was broken down into first level concepts, or major themes, and second-level categories, or sub-themes. For example, interviewees frequently reported difficulty in performing important day-to-day activities such as reading, driving, and playing sports. Difficulty in performing day-to-day activities became a concept or a major theme and the related things (reading, driving, and playing sports) became categories, or sub-themes (Fig. 1). One of the authors (MP) did the coding. Once the coding was completed, the concepts and the categories were re-assessed by the authors (JK & KP) to decide whether they formed major or sub themes. Any discrepancies between the authors were resolved by discussion. Comparison within and between the two groups (hereditary retinal diseases vs acquired retinal diseases) were carried out based on number of issues (i.e. coded by nodes) identified across common themes. The qualitative software program QSR NVivo 11 (QSR International Pty Ltd) was used to systematically code the transcripts.

## Results

Seventy-nine semi-structured interviews were conducted with participants with hereditary retinal disease (*n* = 32) and acquired retinal diseases (*n* = 47). In the acquired retinal diseases group, 80 participants were approached, 15 participants declined, 13 participants were excluded from the study (could not speak English = 6, hearing loss = 4, had multiple ocular comorbidities = 3) and 5 did not show interest in participating in the study. In the hereditary retinal diseases group, 41 participants were approached and 9 declined to participate. Participants in the hereditary retinal diseases group were younger, mostly working, had bilateral eye diseases and more visually impaired compared to the participants with acquired retinal diseases who were older, had unilateral eye disease, and were mostly retired and less visually impaired (Table 1). The hereditary retinal diseases group comprised of retinitis pigmentosa (*n* = 23), cone dystrophy (n = 2) and macular dystrophy (*n* = 7) and the acquired retinal diseases group comprised of vascular occlusions (*n* = 18), epiretinal membrane (*n* = 20) and macular hole (*n* = 9).

**Table 1:** Socio-demographic details of the study population

Variable	Hereditary retinal diseases *n* = 32	Acquired retinal diseases *n* = 47
Age (years, *n* (%))		
> 55	19(59)	44(94)
Median age, IQR	57, 44 to 69	73, 65 to 78
Range	28 to 81	34 to 90
Median age of onset of disease, IQR (years)	18, 12 to 31	70, 62 to 75
Duration of the disease (years), *n* (%)		
Less than 5 years	3(9)	31(66)
5 to 10 years	1(3)	14(30)
More than 10 years	28(88)	2(4)
Gender, n (%)		
Female	20(63)	29(62)
Main language spoken, *n* (%)		
English	29(91)	42(89)
Other	3(9)	5(11)
Marital status, *n* (%)		
Married	19(59)	15(32)
De facto/ divorced/separated/widowed	8(25)	27(57)
Never married	5(16)	5(11)
Education level, *n* (%)		
Secondary or less	10(31)	34(72)
TAFE/university degree	22(69)	13(28)
Employment status, *n* (%)		
Working	20 (63)	5(11)
Visual acuity (worse eye), *n* (%)		
Better than 6/18	3(9)	21(44)
6/18 to 6/60	17(53)	20(43)
Less than 6/60	11(34)	6(13)
Laterality, *n* (%)		
Bilateral	32(100)	6(13)
Ocular comorbidity, *n* (%)		
Yes	12(38)	16(34)
Medical comorbidity, *n* (%)		
Yes	16(50)	28(60)

Percentage of some variables may not be equal to 100% due to missing data

We identified nine quality of life themes (domains) relevant to both the groups. The themes were: (1) difficulty in performing important day-to-day activities (activity limitation), (2) facing emotional and psychological challenges (emotional well-being), (3) struggle with social interaction (social well-being), (4) having a myriad of ocular and visual symptoms (symptoms), (5) concerns about health, disease outcome and personal safety (health concerns), (6) problems with mobility and orientation (mobility), (7) inconveniences associated with eye condition (conveniences), (8) effect on work and finance (economic), and (9) coping with the eye condition (coping). These themes were further synthesized to identify whether they could emerge as important domains of ophthalmic quality of life. These themes conform to the existing ophthalmic quality of life domains proposed by our group.

**Fig. 2:**
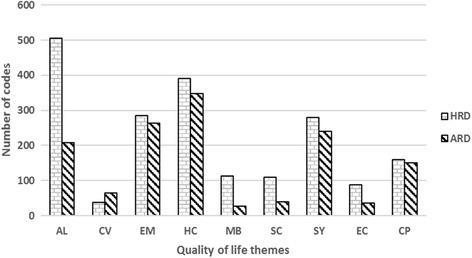
Quality of life (QoL) themes/domains in hereditary retinal diseases (HRD) and acquired retinal diseases (ARD). Codes = number of times the issue was discussed across all the transcripts analyzed. X-axis represents QoL themes/domains and Y-axis represents number of coded segments for each QoL theme/domain. AL, activity limitation; CV, convenience; EM, emotional well-being; HC, health concerns; MB, mobility; SC, social well-being; SY, symptoms; EC, economic; CP, coping

Generally, the hereditary retinal diseases group had expressed more issues (denoted by number of coded segments) across all domains except one than the acquired retinal diseases group (Fig. 2). Activity limitation was the most prominent quality of life issue among participants with hereditary retinal diseases and health concerns was the most prominent quality of life issue among participants with acquired retinal diseases (Fig. 2). We compared the quality of life issues between the two groups to identify common and unique issues. Common and unique issues were based on the iteration. Common issues are quality of life issues that were reported in both the groups and unique issues were those, which were reported in only one group. Within the groups, the quality of life issues were similar. More than 80% of the quality of life issues were common between retinitis pigmentosa, cone dystrophy and macular dystrophy and more than 70% of the issues were common between vascular occlusion, epiretinal membrane and macular hole.

However, between the disease groups, some of the quality of life issues were common, but overall; we found that many of the quality of life issues were unique to the disease groups.

The quality of life issues in people with hereditary retinal diseases and acquired retinal diseases are discussed below.

### Theme 1: People with both acquired retinal diseases and hereditary retinal diseases had trouble in performing important day-to-day activities (Activity limitation)

Activity limitation was identified as the major quality of life issue in participants with hereditary retinal diseases (Fig. 2). The major activity limitations were difficulty in reading, driving, seeing in poor lighting conditions, shopping, using computers and playing sports. They reported difficulty in reading small prints, numbers, and labels. Most participants expressed that using large print books or voice-activated books enabled them to continue reading. Difficulties in being able to see at night caused frustrations. Not being able to drive was reported as a big loss as they had to depend on friends or family members for daily commute (Table 2). Frequent re-organizing or re-arranging things in the supermarket and inability to read price tags made shopping a huge challenge. They also reported difficulty in playing outdoor games especially ball games.

**Table 2:** Examples of quotes expressed by the participants with hereditary retinal diseases and acquired retinal diseases

Major quality of life themes	Sub-themes		Hereditary retinal diseases		Acquired retinal diseases
Activity limitation	Reading	Responses = 66 Participants = 22	*“During the teenage years I could read large print. After that I lost the vision to read and I’ve only got light perception now”*	Responses = 21 Participants =12	*“That’s right, I just went and got an eye test and they said ‘you need glasses for reading’ because I can’t read properly so they did bifocals but it wasn’t until later that I was told that my eyesight was – something wrong with it”*
	Seeing in different light conditions	Responses = 55 Participants = 21	*“I really can’t do much by myself, I need someone to help me around at night”*	Responses = 9 Participants = 5	*“I can still see at night with the headlights on but I don’t go out much at night.”*
	Driving	Responses = 42 Participants = 16	*“The first manifestation was when I was driving I couldn’t see the white lines on the road and I was trying to share lanes with other car drivers”*	Responses = 37 Participants =17	*“I see nothing hardly but my left eye is good so - but with driving and that I find, you know, I’ve got to turn my head right around or else I don’t see what’s coming from my right”*
	Shopping	Responses = 49 Participants = 22	*“I really do feel that my sight now is impacting on my independence so being able to go out and shop on my own is becoming extremely difficult to do”*	Responses = 8 Participants = 7	*“Another thing is when you go to the supermarket and you stand at the top of the aisle and look down you can’t read what the products are so you’ve got to walk up and down each one looking at the - but minor things, minor things for me.”*
Emotional well-being	Feel frustrated	Responses = 45 Participants =15	*“It’s frustrating because you want to do what everybody else can do and sometimes it’s the little things that becomes most frustrating”*	Responses = 24 Participants = 9	*“Probably the clinics are very busy and you’re there for quite a while sometimes. There’s days where I’m in there for three to four hours and it can get very frustrating. Just sitting around for three or four hours, it’s very frustrating and you get very tired and you just want to get in and get out.”*
	Feel anxious	Responses = 29 Participants = 13	*“Being able to go places that I’m not familiar with on my own, I find that difficult until I get quite anxious about it now because I know how hard it is”*	Responses =16 Participants =14	*“I suppose any anxiety I have might just be that the injections don’t work as well as they were hoping them to because I have already had eight and originally they said normally with these injections you get about six to eight and then hopefully it’s working by then but with mine no shunts were really formed to drain my eye as they were hoping it would.”*
	Feel hopeful	Responses = 18 Participants = 5	*“That wouldn’t worry me because I haven’t got much vision as it is. The only thing I can lose now is light perception and that’s it. I just hope I don’t lose that but that’s about all I can lose.”*	Responses = 47 Participants =16	*“Well I am hoping that when I do have the laser treatment, that I’ll notice a real difference and the strength of my glasses will be reduced. I just feel I will notice a difference. I won’t have those floaties and things.”*
	Feel shocked	Responses = 20 Participants =16	*“It was frustrating because when they give you the diagnosis that you’ve got this eye condition and you’re going to go blind you’re in so much shock and you don’t really know what to do. What probably would have helped would have been one of the staff to say ‘look, here is a bunch of information.”*	Responses = 22 Participants = 11	*I got a terrible shock when I tried to read an eye chart because, as I say, looking with two eyes you can’t notice any difference.”*
Social well-being	Interacting socially with people	Responses = 37 Participants = 20	*If I’m out with people and in a bunch of people, even people I know, and they’re all chattering and I can’t see which one is which and I can’t see which one is talking to me and that, yes, I feel isolated.*	Responses = 7 Participants = 6	*“No, not because of that, because my friends come out home, we have a few drinks and then we go and cook tea, watch TV and go to bed.”*
	Strain in personal relationships	Responses = 5 Participants = 5	*“I was 31 when I was actually diagnosed with RP and that occurred – my marriage had just broken up and I had two children who were seven and nine”*	Responses = 0 Participants = 0	NA
	Getting help and support from your family and friends	Responses = 109 Participants = 24	*When I tried to explain it to my mother and my father – and of course my mother had RP – my father ordered me out of the house and told me he had enough of putting up with his wife for 50 years with RP and he didn’t want another person with RP in the family and told me to get out. No matter what I tried to do I could not make that side of my family understand.*	Responses = 39 Participants = 20	*“No, I’ve told them the full story and my family’s lovely, they’re confident for me and, yeah, they always reassure that everything will be okay and all that sort of stuff”*
	Being part of social activities	Responses = 26 Participants = 15	*Well, yeah, it does because I just can’t do things with – like you’re left out with the parent groups at school and stuff because I can’t get to the coffees; I can’t do the activities everyone else does.*	Responses = 20 Participants = 9	*“I play in a thing called a fun band where we go around and play music at Helping Hand centres and aged care facilities and all of that.”*
Health concerns	Not getting enough information from medical staff	Responses = 55 Participants = 26	*“I had been told by a misinformed medical practitioner when I was in my teens that I would go blind and not be able to see within a few years so my life absolutely turned upside down. That person was wrong and I only found that out in my 30s”*	Responses = 85 Participants = 32	*“He said ‘if you had surgery on your eyes as they are now’ he said ‘you could go blind’ but he wouldn’t tell me why so - excuse the language but I was absolutely pissed off with him.”*
	Bumping into people or objects	Responses = 36 Participants = 20	*“I’ve had a few trips. I fell down some stairs, just two or three stairs, and sprained both ankles”*	Responses = 8 Participants = 6	*“Well I mean I’ve fallen over several times walking down the street and I’ve broken my tooth, broken the front tooth.”*
	People not understanding your visual impairment	Responses = 32 Participants = 8	*“Also people’s perception; people would accuse me of being drunk or on drugs and they didn’t understand.”*	Responses = 2 Participants = 1	*“I have some cousins and they’re concerned, they ask me how it happened and what treatment and stuff so some people understand but then other people – yeah, when you listen to other people’s problems you feel kind of a bit helpless so you don’t really want to ask.*
	Going blind	Responses = 39 Participants =16	*“My biggest fear is that perhaps I will lose it all. I’ve been fighting all these years to retain my vision and my biggest fear is losing it all”*	Responses = 24 Participants =14	*“I was worried that I didn’t – that what they suggested that I have done, I definitely wanted to have the operation because I didn’t want to go blind in my eye and I thought that was most important, to get that fixed if I could”.*
Symptoms	Night blindness	Responses = 68 Participants = 23	*“I haven’t had any night vision for a long, long time.”*	Responses = 0 Participants = 0	NA
	Distorted vision	Responses = 0 Participants = 0	NA	Responses = 26 Participants =11	*“No, it’s just a – just say if I’m looking at a straight line the line’s crooked. It’s not straight, it’s crooked out of my right eye, and I can’t see faces if I’m too far away from people”*
	Restricted field of vision	Responses = 46 Participants = 21	*“Well put it this way, ever since I was young I’ve never had much field of vision; I’ve always had tunnel vision.”*	Responses = 8 Participants = 2	*“You know, sure I could lose my sight altogether with the retina peeling off but they never mentioned that this would affect my peripheral vision, which was as clear as a bell prior to that, and as far as I’m concerned that’s not on”*
	Distinguishing colours	Responses = 28 Participants = 20	*“well, to some extent - but with colour vision I see dark colours as either black or dark blue or dark brown; I can’t differentiate between those colours”*	Responses = 0 Participants = 0	NA
Mobility	Walking around unfamiliar areas	Responses = 34 Participants = 17	*“Being able to go places that I’m not familiar with on my own, I find that difficult until I get quite anxious about it now because I know how hard it is”*	Responses = 0 Participants = 0	NA
	Crossing a street/road	Responses = 4 Participants = 4	*“You know, crossing roads is very difficult.”*	Responses = 5 Participants = 2	*“I have to be careful crossing roads because I can’t see that far up the road to what’s coming”*
	Walking in crowded situations	Responses = 17 Participants = 10	*“I found that I was finding it really difficult in shopping centres and I was starting to avoid going to those places”*	Responses = 0 Participants = 0	NA
	Using steps/stairs	Responses =13 Participants = 9	*“I mean going down steps is the most difficult thing. Ramps are good but steps are not good”*	Responses = 1 Participant = 1	*“When I get off, you know, steps and kerbs and things it’s kind of not where it should be so I have to stop and kind of do it carefully and look where I’m going.”*
Economic	Ability to find employment or get a new job	Responses = 73 Participants = 22	*“I guess it affected my work because I can’t get fulltime work because people don’t want to employ visually impaired people,”*	Responses = 0 Participants = 0	NA
	Costs associated with treatment of the eye condition	Responses = 0 Participants = 0	NA	Responses = 4 Participants = 2	*“No, well, I always basically have to pay – I just have to pay, like anything you get from the chemist really, the Warfarin, it’s like – I think it’s about 13 bucks a bottle or something. They’re just 50 little pills and I usually have to take at least two a day so I suppose that adds up, yeah.”*
	Not being able to work	Responses = 54 Participants = 20	*“I was a nurse and then I was a disability support worker in a mental institution, like in a – what would you call it now – community houses I think they are. I was 2IC in a community house when my vision started to deteriorate so I just - actually I was lucky enough to be able to get a package and leave.”*	Responses = 9 Participants = 5	*“Well actually as my eyes are now I wouldn’t be able to do what I used to do years ago. I wouldn’t be able to do that job now, it’d be too dangerous.”*
	Financial impact from loss of income	Responses = 12 Participants = 8	*“I was earning a very good income and that was cut completely. Well now, as a remedial massage therapist I do have an income but it’s still very small”*	Responses = 5 Participants = 3	*I suppose it has because the work I do, I’m on a casual rate which means when I have to go to hospital I actually don’t get paid at all when I’m not there.*
Convenience	Having to do positioning after surgery	Responses = 0 Participants = 0	NA	Responses = 42 Participants = 15	*“Well after you had the surgery you’ve got to lay on your belly for two weeks and that is absolute murder.”*
	Having to rely on others for help	Responses =37 Participants = 18	*“Not at all. Well, yes, because I’ve got to call on my - I have two children and I’ve got to call on them to read my mail to me and to do some computer work for me sometimes, little things like that”*	Responses = 8 Participants = 8	*“I think the inconvenience was mostly the need for regular visits for anti-VEGF injections because I don’t like driving right after an injection because I’ve got one eye patched and it’s pretty sore and bloodshot and so somebody else goes with me and that – you know, you’re doing that every six weeks for a while and so it’s an inconvenience, not only to me but to somebody else.”*
	Having to plan and organize for the things beforehand	Responses = 8 Participants = 5	*“It’s very hard to be spontaneous with anything. I can’t go down to a far place and have a swim, that would be too big a project now, whereas if I could drive that’s no problem. For me to go to the beach I’ve got to plan ahead, probably going to be a two day thing, so it’s that lack of ability to participate in something spontaneously.”*	Responses = 0 Participants = 0	NA
	The amount of time needed when attending the eye appointment	Responses = 2 Participants = 1	*“It was a morning appointment and I felt like I was there all morning, like it was hours.”*	Responses = 8 Participants = 7	*“Just sitting around for three or four hours, it’s very frustrating and you get very tired and you just want to get in and get out”*
Coping	Trying to be positive	Responses = 28 Participants = 15	*“you know, being blind and alive is better than being young and dead so, no, I’m quite strong about that and I always think on the positive”*	Responses = 12 Participants = 12	*Yes, I probably will then but the way I am now I’m not frightened of anything. You’ve just got to think positive. You start thinking negative you’ll just go backwards.*
	Thinking that there are people much worse than you	Responses = 8 Participants = 8	*“When I go to like to the Blind society and stuff there’s always people so much worse you feel bad complaining”*	Responses = 7 Participants = 6	*“Not until you go down there and see them and there’s a lot of people worse than me”*
	Attributing the eye condition to ageing	Responses = 0 Participants = 0	NA	Responses = 15 Participants = 12	*“My eyes have deteriorated more through age because I’ve just turned 60 so your eyesight is not as sharp.”*
	Accepting the eye condition	Responses = 26 Participants = 11	*“Cry and then pull myself up by my socks and get on with it”*	Responses = 36 Participants = 21	*“Well I didn’t feel anything. I thought ‘oh well, I’ve been told that. That’s what’s wrong. Well, I’ve just got to accept it’. You can’t say ‘oh no, I don’t want it’. It’s not going to go away”*

*NA* not available

In the acquired retinal diseases group, activity limitation was only the fourth biggest issue (Fig. 2). The major activity limitations in this group were difficulty in reading, driving, watching television, and engaging in leisure activities (Table 2). They reported difficulty especially reading fine prints and street/road signs. In contrast to people with hereditary retinal diseases who had difficulty in playing outdoor games, people with acquired retinal diseases had difficulties in playing indoor games such as board games and doing puzzles. In contrast to people with hereditary retinal diseases who could not drive, people with acquired retinal diseases were driving but expressed that driving had become challenging especially at night. As these eye conditions predominantly involved the central retina, they often reported difficulty in recognizing people’s faces.

### Theme 2: Participants with both hereditary retinal diseases and acquired retinal diseases faced emotional and psychological challenges (Emotional well-being)

Participants with both hereditary retinal diseases and acquired retinal diseases expressed positive and negative emotional comments. However, people with hereditary retinal diseases expressed more negative comments than positive comments. The commonly expressed emotional comments in the hereditary retinal diseases group were frustration, anxiety, shock, depression, and anger. There was an inability to do things like others such as to read, to drive and to find a suitable job, which often resulted in frustrations. Having to keep up with the technology and not knowing how their eye conditions were going to progress caused anxiety. They expressed that being diagnosed as legally blind was more shocking than being diagnosed with the eye condition. Uncertainty about the future and having to lose their driving license caused depression.

In contrast to participants with hereditary retinal diseases, participants with acquired retinal diseases were more optimistic about their eye condition. They believed that treatment would make their eye condition better. Participants whose vision had not improved with treatment worried about losing their sight and involvement of the other eye. Having to wait for long hours in the clinics, frequent eye appointments and repeated eye tests were some of the reasons for their frustrations. They feared the repeated eye injections and laser treatments.

### Theme 3: Participating in social activities was problematic (Social well-being)

People with hereditary retinal diseases reported difficulties with social interaction. They experienced more difficulty in getting help and support from friends and family members compared to participants with acquired retinal diseases. Some participants experienced strain in their personal relationship especially with their partners due to their eye condition. Despite the lack of support, many expressed that they overcame the hurdles by learning strategies to be independent. Difficulty in recognizing faces, social cues and body language made them feel isolated in social gatherings. They frequently associated themselves with societies/government organizations to keep themselves updated about their eye condition.

Participants with acquired retinal diseases did not have to rely as much on friends and family members for support. Meeting up regularly with family members and friends and being part of social activity groups such as Facebook groups, church groups and book clubs were some of the social activities among them. The participants shared that they had often discussed their eye condition with their family members to increase awareness.

### Theme 4: Concerns about health and safety were significant (Health concerns)

Health concern was a major issue in both the disease groups and was the major quality of life issue in the acquired retinal diseases group (Fig. 2). Participants in the hereditary retinal diseases group were often concerned about accidents such as falling, tripping, and bashing into things due to their limited peripheral vision. Many of them articulated that their experiences with their specialist were unpleasant, as they felt that their specialists could not understand their visual loss. They often worried about going blind and having to live on their own. Generally, this group of participants felt that their friends and family members did not understand their visual impairment. Not knowing what is going to happen in the future, fear of passing the disease to the kids, and fear of losing their partners were some of the other important concerns in this group.

Most participants in the acquired retinal diseases group were not aware of their eye condition before diagnosis. In most of them, an optometrist diagnosed their eye condition on routine examination. They expressed unhappiness towards their medical service providers who often did not communicate well about their disease/s. Treatment outcomes were the main concern among participants who were undergoing treatments. Participants with treatment failure expressed concerns about the possibilities of disease recurrence.

### Theme 5: Visual symptoms were abundant in both the groups (Symptoms)

Participants in both the disease groups reported a myriad of visual symptoms. Night blindness, restricted field of vision, difficulty in discerning colours and difficulty in light adaptation were the prominent symptoms in retinitis pigmentosa [40] and difficulty with central vision was the prominent symptom in macular dystrophies.

Difficulty with central vision was common to participants with vascular occlusion, epiretinal membrane and macular hole. Individuals with vascular occlusions experienced sudden loss of vision, distortion of vision and seeing floaters. Eye pain and bloodshot eyes were reported after receiving anti-vascular endothelial growth factor eye injections. Participants with epiretinal membrane reported distortion of vision and difficulty in focussing. Participants who have undergone vitrectomy and gas tamponade reported double vision and wobbly vision.

### Theme 6: Problems with mobility and orientation (Mobility)

Mobility was a major issue in participants with hereditary retinal diseases especially in retinitis pigmentosa [40]. They often reported difficulty walking outdoors, walking in a cluttered environment and navigation in unfamiliar places. They also reported difficulty using steps and escalators. Stepping on or off a train or a tram was a challenge. Difficulty in negotiating obstacles while walking and difficulty in navigating in the dark/night were some of the mobility difficulties unique among these participants.

The major mobility difficulties among participants with acquired retinal diseases were crossing a street/road, walking in the dark/night, and walking on uneven grounds. Difficulty in walking on uneven grounds and negotiating bumps/cracks in the path were some unique mobility difficulties in this group.

### Theme 7: Impact on work and finance (Economic)

Work and finance was one of the major issues among the participants with hereditary retinal diseases because most of them were young and working (Table 1). Participants in this group reported that they were unable to pursue the career of their choice. Not being able to get employment often caused fear and anxiety. Lack of mobility and inability to drive restricted their job opportunities. They also reported difficulty in getting help and support from government and other social welfare organizations. Costs associated with looking after guide dogs and attending training courses were some of the other financial implications specific to participants with hereditary retinal diseases.

Participants with acquired retinal diseases had less job-related constraints due to their eye disease as most of them were retired. Some of the financial implications were due to the costs associated with seeing a specialist, costs associated with buying medications and undergoing eye procedures.

### Theme 8: Inconveniences in day-to-day life were very common (Conveniences)

Participants with both hereditary retinal diseases and acquired retinal diseases reported myriad of inconveniences for having to live with their diseases. Between two groups, participants with acquired retinal diseases expressed more inconveniences in their day-to-day life (Fig. 2). Most of the inconveniences in participants with acquired retinal diseases were associated with their treatment. Having to keep face/head position (e.g. face down positioning after vitrectomy) for a prolonged time was reported as a major inconvenience by participants with epiretinal membrane and macular hole. Those individuals with vascular occlusions reported that the major inconveniences were undergoing repeated laser treatment, injections and having repeated eye tests. Long waiting hours in the clinic and having to go for frequent eye appointments were some of inconveniences unique to this group.

The major inconveniences in the hereditary retinal diseases group often resulted from having to depend on others for transportation and travelling by public transport. Not being able to read without assistance was also a major inconvenience. Inability to participate in things spontaneously and losing or misplacing things frequently were some of the other inconveniences.

### Theme 9: Despite all the odds many participants coped well (Coping)

The use of coping strategies to manage the stress of vision loss was common in both the disease groups. Participants with hereditary retinal diseases were coping better compared to participants with acquired retinal diseases. Most of the participants learned to accept their eye condition and maintained a positive attitude. The participants also kept themselves distracted by engaging in useful activities such as listening to audio books, playing sports, and engaging in adventurous activities such as skydiving, skiing and SCUBA diving. Some of them learned to understand the diseases, which helped them to deal with it. Being independent also helped them to get on with life. Seeing other family members adapt to the eye condition also helped them to cope better.

Attributing their eye condition to ageing was a common coping response used by participants with acquired retinal diseases. The other coping responses were trying to ignore their eye condition and indulging in engaging activities such as knitting, reading and gardening. Trusting their doctors, praying, and meditating were some of the unique coping strategies in this group.

## Discussion

Our study revealed that participants with hereditary retinal diseases experience more quality of life issues compared to participants with acquired retinal diseases. Participants with hereditary retinal diseases were more visually impaired compared to participants with acquired retinal diseases and that could be one of the reasons for a greater number of quality of life issues iterated in the hereditary retinal diseases group. The quality of life themes/domains across the disease groups were identical, but when compared with the domains specific issues, they were mostly different. The apparent differences could be due to the differences in the disease in terms of age of onset, duration of the disease, severity of visual loss and employment status. In the hereditary retinal diseases group, the predominant loss of vision was peripheral and binocular, however, in the acquired retinal diseases group; it was mostly central and monocular. The duration of the disease was longer in hereditary retinal diseases and shorter in acquired retinal diseases. Most of the participants in the hereditary retinal diseases group were working and most of the participants in the acquired retinal diseases group were retired (Table 1). Participants with hereditary retinal diseases had severe visual impairment and participants with acquired retinal diseases had only mild to moderate visual impairment. The quality of life issues of people with retinitis pigmentosa has been previously published [40], in this paper we are comparing the quality of life issues of people with hereditary retinal diseases and acquired retinal diseases .

The nine domains were determined from the emerging themes during the analysis. These domains conform to the important ophthalmic domains of quality of life identified in other eye diseases [1, 5, 33, 34]. We found stark differences in types of quality of life issues across these domains between acquired retinal diseases and hereditary retinal diseases. The most prominent quality of life parameter among participants with hereditary retinal diseases was activity limitation, which might be attributed to the fact that participants with hereditary retinal diseases had bilateral eye condition and living with severe visual impairment. On the contrary, health concerns (e.g. concerns of going blind, treatment outcomes etc.) was the prominent quality of life issue in acquired retinal diseases. This could be because most of the acquired retinal diseases are acute and treatable. Similarly, participants with hereditary retinal diseases continuously face progressive loss of vision, which may be one of the reason they express negative emotional comments much more than when compared to positive comments. Frustration, worry, shock, and depression were some of the commonly expressed emotional comments in our study and similar findings were reported in previous studies [41, 42].

Participants with hereditary retinal diseases had more issues with social interaction and mobility compared to participants with acquired retinal diseases. Inability to identify social clues, facial expressions, body language and difficulty in participating in social activities at night affected the social life of participants with hereditary retinal diseases. The mobility issues may be attributable to the loss of the peripheral visual field. Effect on work and finance was an important quality of life theme among participants with hereditary retinal diseases. They had greater economic and financial impacts due to their diseases compared to participants with acquired retinal diseases because most of these participants were working (Table 1). The economic effects may not be part of the health related quality of life but forms a part of quality of life, which is a broader concept than health related quality of life. Participants with hereditary retinal diseases were symptomatic than participants with acquired retinal diseases because hereditary retinal diseases are progressive diseases. Night blindness, progressive visual field loss and difficulty in light adaptation were the common symptoms reported by participants with retinitis pigmentosa in this study [40]. In contrast, a previous study has reported a different set of symptoms (day-to-day visual fluctuations, intermittent diplopia, photopsia, high glare and visual hallucinations) [42].

The type of coping strategies used by an individual depends on the situation they must face. Coping that implies a positive attitude has shown to improve health related quality of life and a passive attitude has shown to worsen the health related quality of life [43, 44]. The coping strategies used by participants with hereditary retinal diseases mostly implied positive attitude (e.g. trying to be positive and acceptance of their eye condition). The coping strategies used by participants with acquired retinal diseases implied passive attitude (e.g. trying not to think about their eye condition and attributing their eye disease to ageing). Participants with hereditary retinal diseases were reported to cope better than participants with acquired retinal diseases as they used positive attitude.

Despite the low prevalence, hereditary retinal diseases and acquired retinal diseases can lead to severe visual impairment and blindness. As new advancements in treatments for hereditary retinal diseases and acquired retinal diseases such as anti-vascular endothelial growth factor injections and gene therapy continue to gain momentum, a comprehensive patient-reported outcome instrument will be invaluable for use in clinical trials to compare the impact of novel treatment modalities from patients’ perspective. However, there are no comprehensive and widely validated patient-reported outcome instrument for these diseases. The way forward is to develop one for each retinal disease. However, it is not feasible to do so because there are too many retinal diseases with low prevalence rate in general population. The best way forward is to lump/split these diseases into groups.

The results of this study provide a scientific basis for splitting vs lumping less common but potentially blinding retinal diseases to develop retina-specific patient-reported outcome instruments. There are several ways of lumping/splitting the retinal diseases. One way is to group them based on the disease pathology into congenital, vascular, infection/inflammatory, trauma and tumours. This type of grouping would create many disease groups and affect the sample size of the groups. The second way is to split them into central retinal diseases and peripheral retinal diseases based on the anatomical location of the disease. The problem with this lumping is that some retinal diseases involve both the central and the peripheral retina and hence may be difficult to group. The third and a simple way would be to lump all the inherited degenerations together and acquired retinal diseases together. Hereditary retinal diseases differ from acquired retinal diseases in the onset, presentation, and manifestation. The division into hereditary retinal diseases and acquired retinal diseases is also supported by our qualitative findings, which compared quality of life issues within and between these two groups. Putting together these findings, we could argue that a single patient-reported outcome instrument would not serve both the disease groups. Therefore, we propose to split other vitreoretinal diseases into two groups for the sake of developing group-specific patient-reported outcome instruments.

This study had some limitations. The method of data collection was interviews and not focus groups. Focus groups are the gold standard method for exploring people’s feelings, motivations, insight, and experience on any topic. As this study involved uncommon retinal conditions, organizing focus groups was difficult. The other limitation was that the hereditary retinal diseases group had fewer people with macular dystrophies and cone dystrophies than retinitis pigmentosa. This could have contributed to some bias in the data interpretation. Having equal number of participants with cone dystrophy and macular dystrophies could have avoided the bias. However, macular dystrophies and cone dystrophies are relatively uncommon inherited retinal disorders and it was difficult to have an equal number of participants with these retinal conditions in this group. Moreover, the acquired retinal diseases group had only participants with vascular occlusions, macular hole and epiretinal membrane and did not have participants with other retinal condition such hemoglobinopathies. This might limit the relevance and generalizability of our findings to all acquired retinal diseases.

## Conclusions

Quality of life are different between the two disease groups, which may be due to the difference in the onset, presentation, and manifestation of the retinal diseases. Hence, these two disease groups would need separate patient-reported outcome instruments to capture group-specific quality of life impact.

## Additional file

Additional file 1: Semi-structured interview guide for hereditary retinal diseases/acquired retinal diseases. (DOCX 19 kb)
