# Supplementary material for: Exploring the quality of life issues in people with retinal diseases: a qualitative study
Source: J Patient Rep Outcomes. 2017 Sep 21;1:15. doi: 10.1186/s41687-017-0023-4 (PMC5934910; doi:10.1186/s41687-017-0023-4)
Supplement: Supplementary file 1 — Semi-structured interview guide for hereditary retinal diseases/acquired retinal diseases. (DOCX 19 kb) [file 41687_2017_23_MOESM1_ESM.docx]

**Additional file 1**

**Semi-structured interview guide for hereditary retinal diseases (HRD) and acquired retinal diseases (ARD)**

**Introduction**

Thank you very much for taking the time to participate in this interview. We are currently interested in finding out how having hereditary retinal diseases / acquired retinal diseases impacts on your life as a whole. This includes what you can do and can’t do, how you feel, relationship with others and specific effects of the treatment you have had. We are basically interested in hearing your views, experiences and opinions. This will help us to better understand the needs of people who have hereditary retinal diseases / acquired retinal diseases.

My role here involves asking questions and listening. I won’t actually be participating in the conversation, instead I would like you to feel free to talk as much as you like in response to the questions. I would like to stress that there are no right or wrong answers and we are most interested in your personal views, opinions and experiences. Therefore, whatever you say and share is right and is extremely important to us.

I’m audio recording this interview because I don’t want to miss any of your comments. However, all the information you will provide will remain confidential. Your name will not be attached to any reports arising from this work.

Do you have any questions? Are we happy to move on?

**Question route**

**Warm up questions**

How long have you had hereditary retinal disease / acquired retinal diseases? Which eye is affected?

**Symptoms**

What sort of visual symptoms do you experience due to your eye condition? (E.g. difficulty in night vision, difficulty in seeing in bright light, distorted or tilted vision, difficult in focussing)

What other unwanted symptoms have you experienced because of your eye condition, for example any discomfort or eye strain?

**Role Performance & Leisure**

Has your eye condition affected your ability to do things in everyday life? (E.g. domestic work, personal care or community duties or employment? If so, how?

Has your ability to engage in leisure or social activities been impacted by your eye problem? If so, how?

Do you have less confidence in undertaking these duties/activities?

**Relationships**

Has your eye condition changed the way you interact with your partner, family and/or friends? In what ways?

OR *(following specific questions)*

Do you require greater assistance from family and/or friends because of your eye problem? With what sorts of things (e.g. help with mail, reading, chores, transport)

Do you feel other people understand your problem?

Do you feel your family & friends give you the support you need?

Do you feel you are a burden on family and/or friends due to your eye condition? If yes, in what way?

**Concerns**

Do you have any concerns due to your eye condition and its treatment? (E.g. effect of the disease on you and your family)

Do you have any other concerns? *Prompt each separately if not answered* –(such as about your eyesight, long term effect of the hereditary retinal disease / acquired retinal disease, personal safety, side effects of the treatment or losing your driver’s licence, relationship with your partner, family members and friends)

What are your views on the quality of medical care received? Do you feel the advice or information received about your eye condition is adequate? Do the medical staffs communicate effectively? Do you feel your problems are understood?

**Emotions**

How did you feel when you were first diagnosed with hereditary retinal disease / acquired retinal disease?

How have the impacts of the symptoms and treatment of hereditary retinal disease / acquired retinal disease made you feel? (Feeling of reduced independence, loss of enjoyment, loss of identity or reduced self-esteem)

Do you have any fears and anxieties in relation to your eye condition? (E.g. disease progression, treatments, about the future?)

What are your feelings like in the lead up to appointments, during examination, measurements and treatments?

How do you feel after an appointment? Are you uncomfortable about hearing whether your eye condition is stable or getting worse?

How do you feel after finding your eye condition has become worse, if that has been the case?

How does the knowledge that hereditary retinal diseases / acquired retinal disease can potentially make you legally blind make you feel?

Do you feel depressed or unhappy at times because of your eye condition? If so, can you identify what triggers that make you feel depressed or unhappy?

What do you do to help cope with any negative feelings? Does this help?

**Psychological**

Has your eye condition altered the way you view yourself. If so, how has this changed?

Do you feel that you are in control of your life living with hereditary retinal disease / acquired retinal disease?

*Have you had any issues with …………….(prompt separately)* because of you eye condition and its treatment/s?

- - Identity, self-image
  - Self-esteem, confidence
  - Loneliness/ isolation
  - Coping
  - Others

**Inconveniences**

In your experience, what are the major inconveniences associated with having hereditary retinal disease / acquired retinal disease and its treatment? (Prompts: what about appointments, routine eye tests, instruments used to assess your eyes, time taken, travel, parking)

**Costs**

How does having hereditary retinal disease / acquired retinal disease and its treatment affect you financially?

Do you feel there have been direct or indirect costs associated with your eye condition and its treatment? (E.g. travel, health insurance, loss of income)

How does having hereditary retinal disease / acquired retinal disease affect your work life?

**Treatment/Medical Care**

What sort of treatments do you currently receive or have previously received for your eye condition? How have these treatments affected you? (E.g. day-to-day activities, emotionally, financially)

What are your expectations for your treatment? Do you find your expectations are met?

What are your views on the quality of medical care received? Do you feel the advice or information received about hereditary retinal diseases is adequate? Does the medical staff communicate effectively? Do you feel your problems are understood?

**Everyday Tasks (Activity limitations)**

Do you experience problems performing certain tasks? What are they? (E.g. reading at close/distance, writing, watching television, driving, recognising faces and objects, household chores)

Do you have trouble finding things in shops? Reading price tags?

Are you aware of any tasks you can no longer perform?

**Mobility**

Do you find that you have more difficulty getting around? What kind of difficulties have you experienced? (E.g. crossing roads, going up and down stairs, visiting friends or neighbours)

Do you feel you have more accidents or you bump into things more often? Can you describe such instances?

Is transportation an issue? If so, how?

**Low vision aids**

Do you use any visual aids? Do you mind using them? Do they help? (E.g. magnifying glasses, lamps, books with large print, audio books)

**Summary**

Thank you for your contribution to this project. I highly appreciate for your time and patience. Your experience will be very helpful to us in further understanding how hereditary retinal diseases / acquired retinal disease and its treatment impacts on patients’ lives.
